# Exploring the genetic architecture of powdery mildew resistance in wheat through QTL meta-analysis

**DOI:** 10.3389/fpls.2024.1386494

**Published:** 2024-07-03

**Authors:** Divya Sharma, Neeraj Budhlakoti, Anita Kumari, Dinesh Kumar Saini, Anshu Sharma, Aakash Yadav, Reyazul Rouf Mir, Amit Kumar Singh, V. K. Vikas, Gyanendra Pratap Singh, Sundeep Kumar

**Affiliations:** ^1^ Divison of Genomic Resources, ICAR-National Bureau of Plant Genetic Resources, New Delhi, India; ^2^ Centre for Agriculture Bioinformatics, ICAR-Indian Agricultural Statistics Research Institute, New Delhi, India; ^3^ Department of Botany, University of Delhi, Delhi, India; ^4^ Department of Plant Breeding and Genetics, Punjab Agricultural University, Punjab, Ludhiana, India; ^5^ Department of Genetics and Plant Breeding , Sher-e-Kashmir University of Agricultural Sciences & Technology of Kashmir (SKUAST-K), Srinagar, Kashmir, India; ^6^ Divison of Crop Improvement, ICAR-Indian Agricultural Research Institute, Regional Station, Wellington, Tamilnadu, India

**Keywords:** wheat, mQTL, candidate gene, confidence interval, GWAS, consensus map

## Abstract

Powdery mildew (PM), caused by *Blumeria graminis f.* sp. *tritici*, poses a significant threat to wheat production, necessitating the development of genetically resistant varieties for long-term control. Therefore, exploring genetic architecture of PM in wheat to uncover important genomic regions is an important area of wheat research. In recent years, the utilization of meta-QTL (MQTL) analysis has gained prominence as an essential tool for unraveling the complex genetic architecture underlying complex quantitative traits. The aim of this research was to conduct a QTL meta-analysis to pinpoint the specific genomic regions in wheat responsible for governing PM resistance. This study integrated 222 QTLs from 33 linkage-based studies using a consensus map with 54,672 markers. The analysis revealed 39 MQTLs, refined to 9 high-confidence MQTLs (hcMQTLs) with confidence intervals of 0.49 to 12.94 cM. The MQTLs had an average physical interval of 41.00 Mb, ranging from 0.000048 Mb to 380.71 Mb per MQTL. Importantly, 18 MQTLs co-localized with known resistance genes like *Pm2*, *Pm3*, *Pm8*, *Pm21*, *Pm38*, and *Pm41*. The study identified 256 gene models within hcMQTLs, providing potential targets for marker-assisted breeding and genomic prediction programs to enhance PM resistance. These MQTLs would serve as a foundation for fine mapping, gene isolation, and functional genomics studies, facilitating a deeper understanding of molecular mechanisms. The identification of candidate genes opens up exciting possibilities for the development of PM-resistant wheat varieties after validation.

## Introduction

Powdery mildew (PM), caused by the obligate biotrophic fungus *Blumeria graminis*is *f.* sp. *tritici (Bgt*), is a widespread global disease resulting in substantial wheat yield losses. Over the past forty years, there have been numerous global outbreaks of wheat PM (Morgounov et al., 2012). The pathogen is the sixth most significant fungal disease of wheat (Dean et al., 2012) and the eighth largest cause of wheat production loss worldwide (Savary et al., 2019). Generally, PM has predominantly been observed in regions characterized by ample rainfall and humid climate (Bennett, 1984). Nonetheless, its emergence as a significant concern in drier and warmer regions is leading to substantial reductions in wheat yield in these areas. This shift could be attributed to the cultivation of semi-dwarf wheat varieties, the increased use of nitrogen-based fertilizers, and the practice of dense planting in the field (Wang et al., 2005; Morgounov et al., 2012). Resistance towards PM is genetically inherited in wheat crop and is controlled by both race-specific and non-race specific genes conferring a qualitative and quantitative resistance. Race-specific resistance is largely heritable, conferred by a single resistance gene, and provides complete resistance to some specific pathogen infections but not others. Non-racial resistance provides a form of partial resistance that is not reliant on specific pathogen avirulence genes. This type of resistance permits infection but limits the spread of the disease (Maurya et al., 2021; Cheng et al., 2022; Mapuranga et al., 2022c).

Most PM resistance research has concentrated on key genes that are thought to be qualitatively race-specific. For instance, extensive research is conducted on the *Pm3* resistance gene and its various alleles due to their ease of manipulation, transient expression, simple inheritance, and ability to confer complete resistance. This resistance is often associated with the hypersensitive response, but it is effective only against a small number of pathogen races and can be readily overcome by new virulent pathogen strains (Yahiaoui et al., 2004; Koller et al., 2018; Simeone et al., 2020). Until recently, the primary emphasis in plant studies has been on adult-plant resistance, which is associated with long-lasting and non-specific resistance. This resistance involves the interplay of multiple genes that delay and obstruct fungal infection, growth, and reproduction during the adult-plant phase (Jakobson et al., 2012). Therefore, combining multiple resistance genes is considered as the most economical and environmentally friendly strategy for increasing the persistence of resistance against most fungal infections in wheat. The attainment of this goal necessitates the integration of conventional breeding methods with molecular techniques, offering the potential to enhance the selection efficiency for resistance to PM and traits related to crop yield (Bapela et al., 2023).In recent decades, the introduction of Next-Generation Sequencing (NGS) technology for genotyping and advancements in genomic-assisted breeding has significantly expedited the identification and incorporation of genes for PM resistance into commercially grown wheat varieties. Moreover, 19 PM resistance genes/alleles have been cloned, e.g., *Pm1a*, *Pm2*, *Pm3* (*Pm3a to Pm3g, Pm3k*, and *Pm3r*), *Pm4*, *Pm8*, *Pm17*, *Pm21*, *Pm24*, *Pm38/Yr18/Lr34/Sr57*, *Pm46/Yr46/Lr67/Sr55*, *Pm60* and *WTK4* (Yahiaoui et al., 2004; Srichumpa et al., 2005; Bhullar et al., 2009; Cao et al., 2011; Hurni et al., 2014; He et al., 2018; Hewitt et al., 2021; Sánchez-Martín et al., 2021; Gaurav et al., 2022). Of these, only *Pm3k* belongs to tetraploid wheat (Yahiaoui et al., 2009). Most of these genes have been incorporated into wheat from closely related species and their wild relatives. However, due to their reduced resistance levels and the undesirable linkage drag they carry, these genes have not seen widespread commercial utilization (Friebe et al., 1994).

Various research groups have previously developed high-density linkage maps and used them in QTL mapping studies for PM resistance (Singh et al., 2022). Nonetheless, the majority of the QTLs identified in different research studies have not undergone fine mapping due to overlapping genomic regions and have seldom been utilized in marker-assisted breeding programs. This appears to be due to variations in experimental designs, environmental conditions, genetic backgrounds of the parental strains, population sizes, types, and densities of molecular markers employed, as well as the statistical methodologies employed in subsequent analyses. Moreover, when focusing on wheat specifically, additional factors come into play, including the complexity of the hexaploid wheat genome, the prevalence of highly repetitive sequences within the genome, and the lack of comprehensive high-density linkage maps (Kumar et al., 2023).

Meta-analysis of QTLs retrieved from different independent studies, is an alternate method that can help in precise mapping of traits (Sharma et al., 2023). MQTL analysis is a relatively new concept and is rapidly emerging an efficient method for narrowing the confidence intervals (CI) of overlapping QTLs, allowing for rapid and efficient discovery of candidate markers and genes linked to the trait of interest (Kumari et al., 2023; Sharma et al., 2023). Meta-analysis has already been performed for various traits in wheat (Kumar et al., 2021; Pal et al., 2021, Saini et al., 2022b) including resistance to different diseases such as leaf rust (Soriano and Royo, 2015; Amo and Soriano, 2022), stem rust (Yu et al., 2014), tan spot (Liu et al., 2020), Fusarium head blight (Liu et al., 2009; Löffler et al., 2009; Venske et al., 2019; Zheng et al., 2021), stripe rust (Jan et al., 2021; Kumar et al., 2023), multiple disease resistance (Pal et al., 2022; Saini et al., 2022a) and PM resistance (Marone et al., 2013). The most recent study on PM resistance, published in 2013, conducted a meta-analysis with a dataset consisting of only 96 QTLs and identified 24 MQTLs (Marone et al., 2013). Since considerable number of QTLs for parameters contributing to PM resistance have been reported after this last report of MQTL analysis for PM resistance in wheat, the present study involving MQTL analysis was conducted (based on QTL studies published until July 2021) to supplement the list of MQTLs and candidate genes reported in the earlier MQTL study for PM resistance. Overall, the primary aim of this study is to investigate the genetic basis of PM resistance by identifying promising genomic regions and candidate genes using the newly available wheat genome data, integrating it with GWAS information and analyzing the roles of the identified candidate genes in various wheat tissues. The outcomes of this research will have significant utility for wheat breeders, providing valuable resources for enhancing resistance to PM in wheat varieties.

## Materials and methods

### Literature survey for collection of QTLs for powdery mildew

We conducted a comprehensive search for articles that reported QTLs linked to PM resistance in wheat, spanning from 1996 to 2021. This search was performed using Google Scholar (https://scholar.google.com/) and other accessible data sources. An additional resource for this search was a recently created Wheat QTL database (http://wheatqtldb.net/; Singh et al., 2022). Each QTL mapping study was screened to extract the following information: (i) mapping population type (e.g., F_2:3_, RILs, DH, BC) and their parents, (ii) population size, (iii) chromosome number, (iv) position of the QTLs and flanking markers, (v) logarithm of odds (LOD) values and (vi) variance explained by the individual QTLs (PVE) and (vii) different disease resistance parameters, such as area under disease progression cure (AUDPC), infection type (IT), maximum disease severity (MDS) and vernalized seedling score (VSS). QTLs with missing data were excluded from the meta-analysis.

### Development of a comprehensive consensus map

The R package called ‘LPmerge’ (Endelman and Plomion, 2014) was used to construct the consensus map following the steps described recently (Kumar et al., 2023). The following genetic maps were employed as the framework maps in this process: (i) the ‘ITMI_SSR map,’ containing 1406 loci (Song et al., 2005); (ii) the ‘Wheat, Consensus SSR, 2004’ map, which includes 1235 marker loci (Somers et al., 2004); (iii) an integrated map for durum wheat with 30,144 markers (Maccaferri et al., 2015); and (iv) the ‘Illumina iSelect 90 K SNP Array-based genetic map,’ comprising 40,267 loci (Wen et al., 2017). For the construction of the consensus map, markers flanking individual QTLs were also included. The genetic maps contained several common markers located at different genetic positions, and these markers were taken into consideration during the construction of the consensus chromosomes maps.

### QTL projection on the consensus map

The genetic map file and the QTL information file from each study were compiled and employed as input text files for QTL projection through BioMercator V4.2 (Sosnowski et al., 2012). This software requires a set of distinct descriptors for each QTL such as the genetic position of the QTL (both peak position and CI), LOD score, PVE value, the trait linked with the QTL, and the size of the mapping population used to identify the QTLs. The QTLProj command of the software was used to homothetically project the peak positions and confidence intervals of each individual QTL onto the consensus map (Gudi et al., 2022). If a specific study did not provide the CI for a particular QTL, we employed population-specific formulas to calculate the 95% CI as follows:


$${\text{For the F}}_{2:3}\text{ and Backcross populations},\text{CI}\left(95\%\right)=530/\left(R^{2}\times \;N\right)$$

$$\text{For the RIL population},\text{CI }\;\left(95\%\right)=163/\left(R^{2}\times N\right)$$

$$\text{For the DH population},\text{CI }\left(95\%\right)=287/\left(R^{2}\times N\right)$$

Where R^2,^is the phenotypic variance explained by the individual QTL.

N is the population size.

### Meta-analysis of the QTLs

QTL meta-analysis for individual wheat chromosomes was conducted using BioMercator v4.3.2 (Goffinet and Gerber, 2000; Veyrieras et al., 2007). Two different approaches were used, while conducting the analysis, depending upon the number of QTLs projected on each chromosome. We followed the method proposed by Goffinet and Gerber (2000), when the count of projected QTLs per chromosome was 10 or fewer and we applied the method outlined by Veyrieras et al. (2007) when the number of QTLs per chromosome exceeded 10. In the initial approach, the selection of the best model was determined by examining the model with the lowest Akaike information criterion (AIC) scores. In the second approach, the optimal model was identified from a range of models, which encompassed the Bayesian information criterion (BIC), AIC, corrected AIC, AIC3, and average weight of evidence (AWE). If a model met the criteria of having the lowest score in at least three of the other models, it was considered the most suitable choice.

### Candidate gene discovery within the MQTLs and their comparison with GWAS-MTAs

For identifying the genes underlying the MQTL regions, the sequences of the markers flanking the MQTL regions were retrieved from public data repositories such as Grain Genes (https://wheat.pw.usda.gov/GG3) or Cereals DB (https://www.cerealsdb.uk.net/cerealgenomics/CerealsDB/indexNEW.php) databases. To ascertain the physical locations of markers, we conducted BLASTN searches against the Wheat Chinese Spring IWGSC RefSeq v1.0 genome assembly, available on the Ensembl Plants database (http://plants.ensembl.org/index.html). The “JBrowse-WHEAT URGI database” (https://urgi.versailles.inra.fr/jbrowseiwgsc/) was also used to determine the physical locations/genomic co-ordinates of specific SNP markers. The peak positions of the MQTLs were calculated using the following formula proposed by Saini et al. (2022a). Furthermore, some high-confidence MQTLs (hcMQTLs) were chosen and investigated for the identification of available CGs. The following criteria was used to choose these hcMQTLs- (i) involvement of at least 3 initial QTLs, (ii) LOD score ≥ 3 and (iii) PVE value >10. The ‘BioMart’ tool, accessible in the Ensembl Plants database, was utilized to retrieve gene models located within a 2 Mb genomic region (1 Mb region on either side of the MQTL peak position). From the InterPro database (https://www.ebi.ac.uk/interpro/), functional annotations for the available gene models were obtained. The genes were further narrowed down based on their Knet scores (Knetminer.com). Additionally, to confirm the effectiveness of the MQTLs, data on PM resistance from 11 GWAS published between 2017 and 2023 were collected and used. The details of these GWAS studies with respect to the population size, type of wheat, platform used for genotyping the mapping panel are given in **Supplementary Table S5**. To pinpoint the significant SNPs and/or marker-trait associations (MTAs) as reported in these GWAS studies, their physical locations were determined using methods such as BLASTN searches, referencing databases, or consulting the source papers. This process was similar to how the physical locations of MQTLs were determined. The MTAs identified through GWAS within 5 Mb genomic areas nearby a MQTL were considered to be co-located. This is because wheat exhibits an extensive linkage disequilibrium (LD) decay range, which is approximately 5 Mb (Yang et al., 2021).

### Identification of known major resistance genes co-localizing with MQTLs

The nucleotide sequences for previously characterized PM genes or sequences of markers linked to these genes were retrieved from databases such as Grain Genes and NCBI (www.ncbi.nlm.nih.gov). Subsequently, BLAST searches were performed using these sequences against the wheat reference genome available in the Ensembl Plants database. After identifying the physical locations of the genes, their positions were compared with the physical coordinates of the MQTLs to determine whether they co-located with the MQTLs.

## Results

### Distribution of QTLs associated with powdery mildew on wheat genome

**Figure 1:**
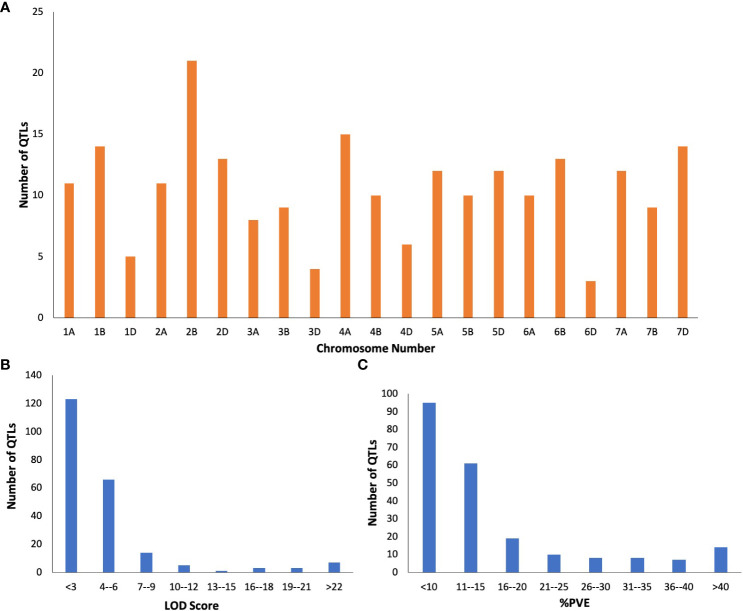
Basic characteristics of QTLs associated with PM resistance **(A)** chromosome-wise distribution of QTLs, **(B)** LOD scores of QTLs, **(C)** % PVE of the QTLs.

QTLs from 34 individual mapping studies (published from 1996 to 2021) were collected and screened for information related to different types of mapping population used, chromosome number, marker positions, LOD score and PVE values. A total of 222 QTLs from PM resistance traits were available for meta-QTL analysis. The detailed information on these QTLs is given in **Supplementary Table S1**. The number of QTLs present on individual chromosomes varied from a minimum of 3 QTLs on chromosome 6D to a maximum of 21 QTLs on 2B (**Figure 1A**). Moreover, the distribution QTLs across the three sub-genomes displayed significant differences, with 79 QTLs located on sub-genome A, 86 on sub-genome B, and 57 on sub-genome D. LOD scores for individual QTLs ranged from 2.1 to 82.6 with an average of 11.3. Most QTLs (83.3% of the total) had their LOD score of< 6 (**Figure 1B**). The phenotypic variation explained by an individual QTL varied from 2.3 to 90% with an average of 16.4%. Approximately, 36.93% of QTLs exhibited a PVE value of< 10% and only a small fraction (6.75%) had a PVE value of > 40%, which suggests the involvement of both major and minor QTLs governing PM resistance (**Figure 1C**).

### Wheat consensus map and QTL projection

**Figure 2:**
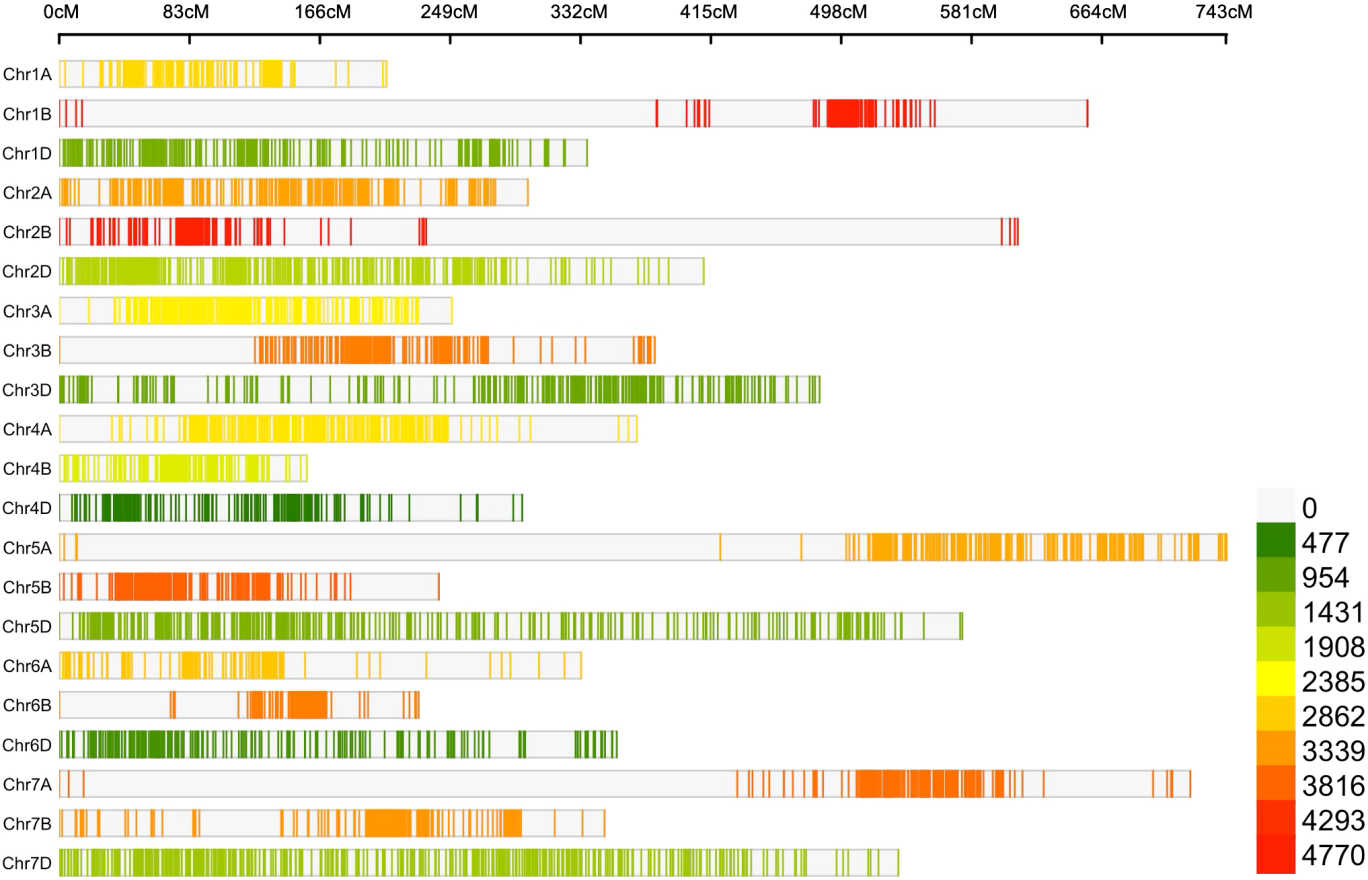
Distribution of marker on the consensus map used in MQTL analysis in the current study.

The high-density Wheat_Consensus_Map_2023 generated in the present study using four different genetic maps exhibited a huge variation in the distribution of markers. The marker density on individual chromosomes varied from 1.37 markers/cM on chromosome 4D to 15.92 markers/cM on chromosome 5B, with a mean of 6.40 markers/cM throughout the genome. The marker density on sub-genome B was highest (9.91 markers/cM) followed by sub-genome A (7.21 markers/cM) and sub-genome D (2.54 markers/cM). The length of individual chromosomes also varied significantly (ranged from 157.78 cM for chromosome 4B to 743.48 cM for chromosome 5A with an average of 406.28 cM). The cumulative genetic map length of all the chromosomes in the map was 8531.99 cM spanned by 54,672 markers. On average, there were approximately 2,603 markers mapped per chromosome and the number of different genetic markers that were mapped on a single chromosome ranged from a few hundred (400 on chromosome 4D) to several thousand (4,769 on chromosome 3B) (**Figure 2**).

### Meta-analysis of QTLs associated with powdery mildew resistance

**Figure 3:**
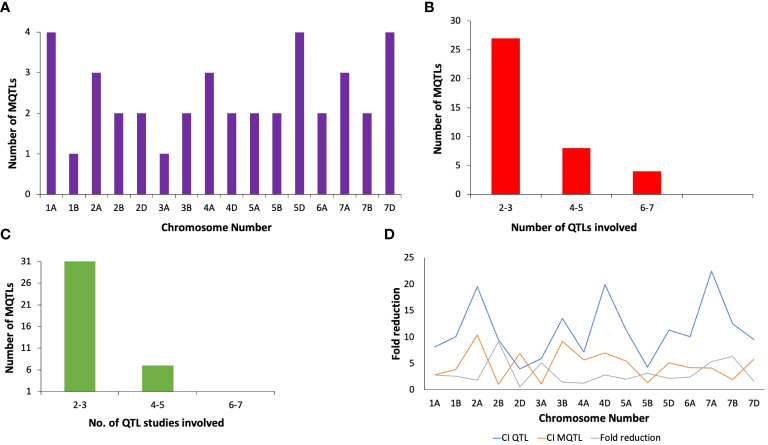
Basic characteristics of MQTLs associated with PM resistance **(A)** chromosome wise distribution of MQTLs, **(B)** the number of QTLs involved in different MQTLs, **(C)** the number of QTL studies involved in different MQTLs, **(D)** fold reduction in confidence intervals of QTLs after meta-analysis.

The method proposed by Gerber and Goffinet (Goffinet and Gerber, 2000) was employed for meta-analysis of QTLs across all wheat chromosomes, except for chromosome 1A, 2A, 2B, 2D, 4A, 5D, and 7D, for which the Veyrieras approach was used (Veyrieras et al., 2007), as these had >10 QTLs projected per chromosome. Out of 222 QTLs, only 168 could be projected (75.6% of the total no. of QTLs) onto the newly constructed consensus map. The remaining QTLs could not be projected because of either of the underlying reasons including (i) lack of sufficient number of shared markers between the consensus and initial genetic maps, and (ii) large CI associated with the initial QTLs. A total of 39 MQTLs were predicted for resistance to PM, consisting of 125 initial QTLs out of the total 168 projected QTLs (**Supplementary Table S2**). The remaining 36 QTLs were singletons (**Supplementary Table S3**) and therefore they were excluded from subsequent analysis. Further, three QTL hotspots were also identified which consisted of initial QTLs from the same studies. These hotspots were located on chromosomes 2B and 2D (**Supplementary Table S4**). Across the three sub-genomes, the maximum number of MQTLs were predicted on sub-genome A (18), followed by sub-genome D (12) and sub-genome B (9). Within sub-genome A, chromosome 1A harbored the highest number of MQTLs (4) while chromosome 3A had the lowest number of MQTLs (1). Similarly, for sub-genome B, chromosomes 2B, 3B, 5B, 6B, 7B comprised of 2 MQTLs each while chromosome 1B comprised of only a single MQTL. For sub-genome D, chromosomes 5D and 7D had 4 MQTLs each which is the highest whereas chromosomes 2D and 4D had 2 MQTLs each **Figure 3A**. No MQTLs were predicted on chromosomes 4B, 1D, 3D and 6D. The number of QTLs per MQTL ranged from ≤ 2 in 16 MQTLs to ≥ 6 QTLs in the 4 MQTLs (viz., MQTL2B.1, MQTL7A.2, MQTL7A.3 and MQTL7D.3) **Figure 3B**.

**Figure 4:**
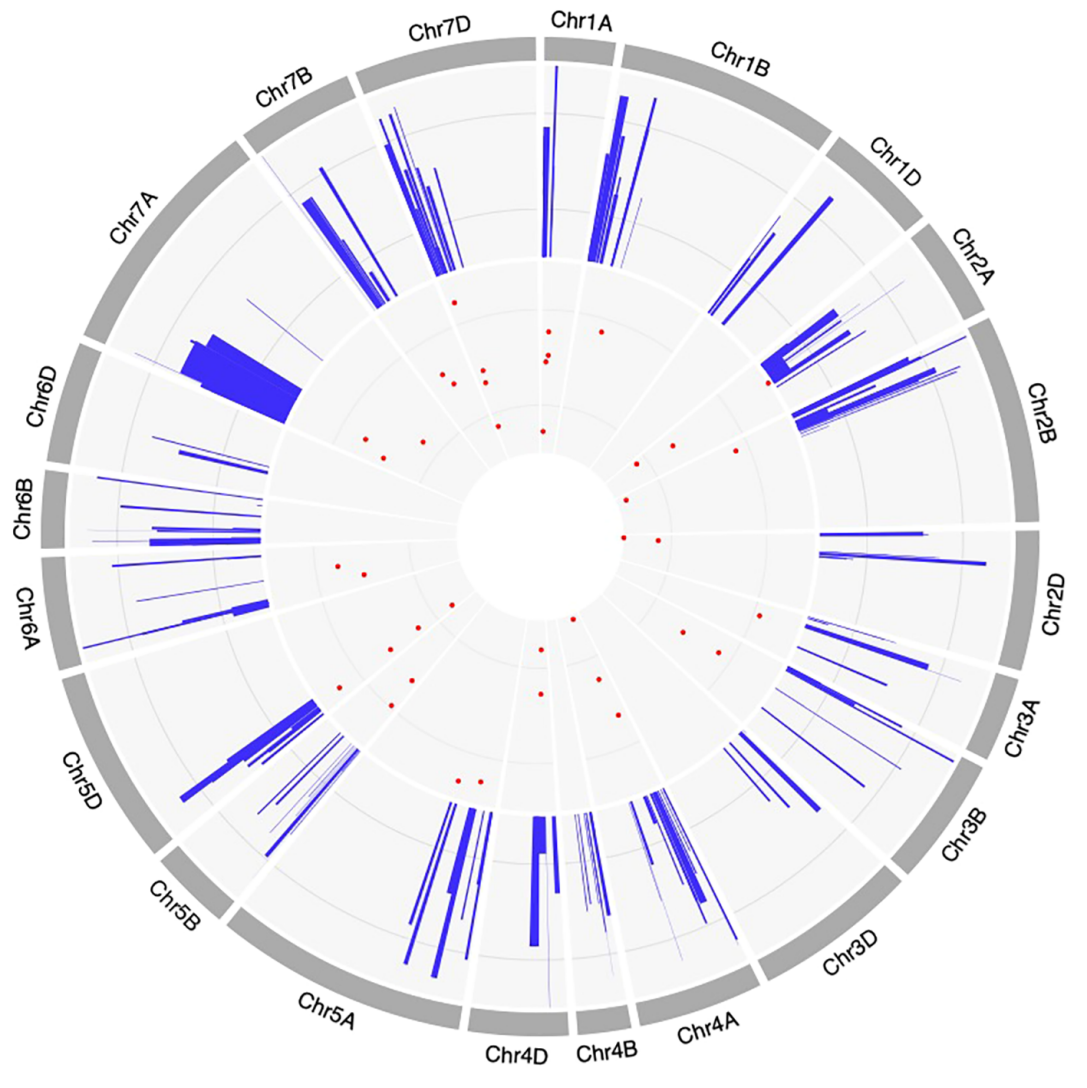
Circos diagram representing the features of QTLs and MQTLs associated with powdery mildew resistance. The information projected includes, (moving inwards) the outermost ring represents consensus map, the positions of projected QTLs on the consensus map, and the innermost ring represents the positions of MQTLs on different wheat chromosomes.

The PVE of individual MQTLs varied from a minimum of 5.9 to a maximum of 76.5% with a mean of 21.17% and the LOD score varied from 1.5 to 30.4 with an average of 6.48. Notable features displayed by the initial QTLs, MQTLs and their distribution across different wheat chromosomes are illustrated in **Figures 1**, **3**, **4**, respectively. The CI of the predicted MQTLs and QTL hotspots varied from 0.06 to 28.14 cM and 0.52 to 1.12 respectively. On an average, the CI of MQTLs and QTL hotspots were significantly reduced by a factor of 2.07 and 1.73 respectively compared to the initial QTLs and there were substantial differences in the extent of CI reduction across different wheat chromosomes (**Figure 3D**). The mean physical CI of the MQTLs was 41.00 Mb, which ranged from 0.000048 Mb (MQTL5B.2) to 380.71 Mb (MQTL6A.2).

### Gene models available in MQTL regions

**Figure 5:**
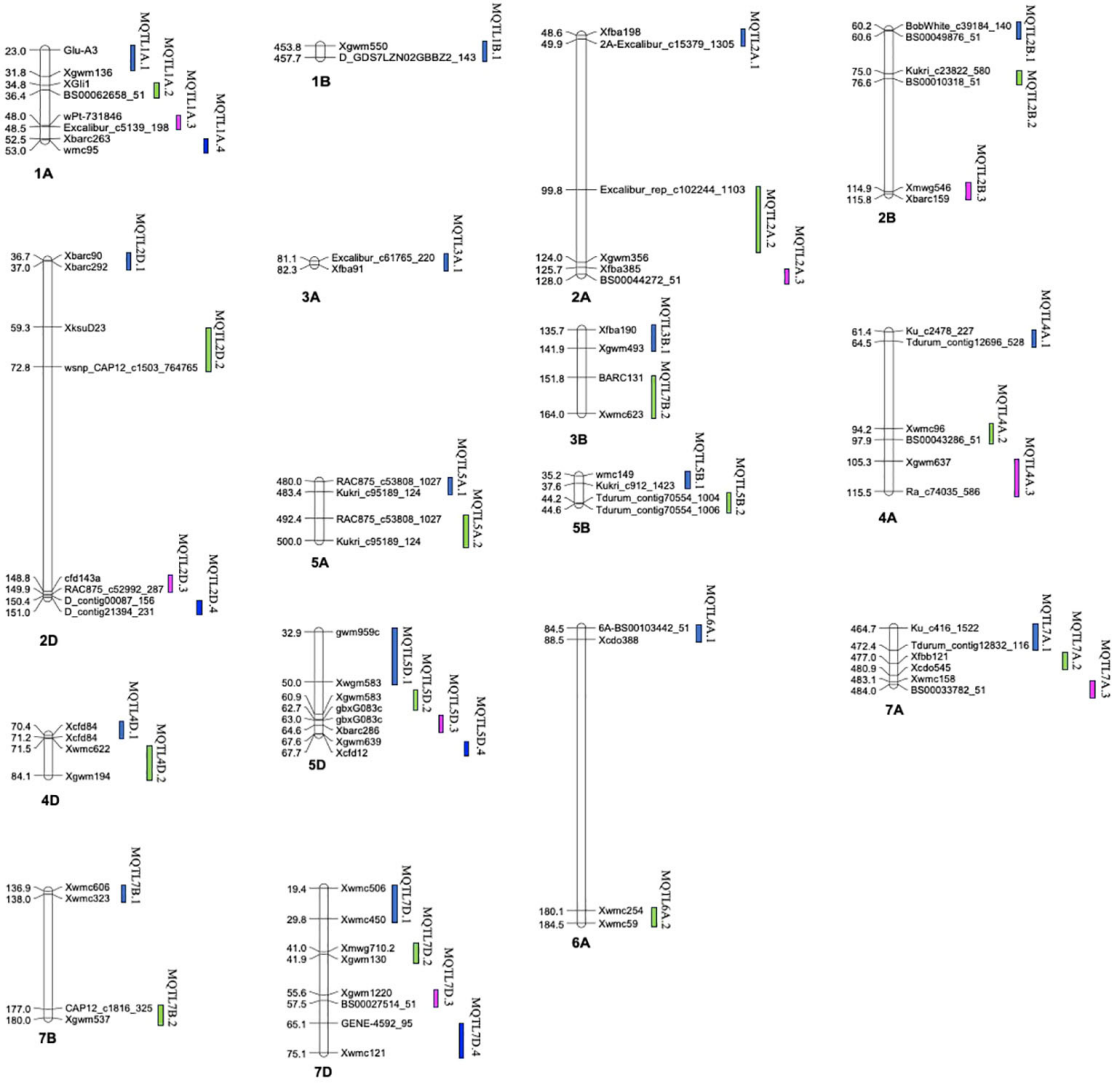
Distribution of MQTLs across the different wheat chromosomes.

A total of 39 MQTLs were mapped to the physical map of the wheat consensus map used in the present study. However, the exact physical positions of three MQTLs (viz., MQTL2A.3, MQTL7A.2 and MQTL7A.3) could not be ascertained due to the unavailability of nucleotide sequences of the markers flanking these MQTLs (**Figure 5**, **Table 1**). To enhance the reliability of the predicted MQTLs, we further refined them, leading to the identification of regions referred to as high-confidence MQTLs (hcMQTLs). In general, each hcMQTL cluster included a minimum of three initial QTLs, with a PVE value greater than 10% and a LOD score greater than three. Further analysis involving gene mining was conducted on 9 hcMQTLs, resulting in the identification of a total of 256 gene models. Among the MQTLs, the one located on chromosome 4D had the maximum number of associated gene models (51). Conversely, the MQTL situated on chromosome 6A had the fewest associated gene models (9) (**Supplementary Table S7**). These gene models encoded different types of proteins, a few of the important ones are as follows-, including (i) NBS-LRR proteins, (ii) transcription factors (TFs) like MADS box and GRAS, (iii) proteins belonging to oxidoreductase class such as cytochrome P450, (iv) proteins with lectin domain, (v) glycoside hydrolases, and (vi) protein kinases.

**Table 1:** MQTLs associated with powdery mildew resistance identified in the present study.

MQTL	Flanking Markers	CI (cM)	LOD Score	PVE%	No. of QTLs involved
MQTL1A.1	*Glu-A3- Xgwm136*	22.98–31.79	5.2	14.4	3
MQTL1A.2	*XGli1- BS00062658_51*	34.78–36.40	10.5	30.2	5
MQTL1A.3	*wPt-731846- Excalibur_c5139_198*	47.98–48.48	2.9	22.9	2
MQTL1A.4	*Xbarc263- wmc95*	52.52–52.95	8.9	46	3
MQTL1B.3	*Xgwm550- D_GDS7LZN02GBBZ2_143*	453.80–457.71	5.8	21.8	4
MQTL2A.1	*Xfba198–2A-Excalibur_c15379_1305*	48.58–49.92	30.4	50.7	4
MQTL2A.2	*Excalibur_rep_c102244_1103- BS00044272_51*	99.84–127.98	12.2	38.2	2
MQTL2A.3	*Xgwm356- Xfba385*	123.95–125.73	8.4	21.9	5
MQTL2B.1	*BobWhite_c39184_140- BS00049876_51*	60.15–60.64	9.1	20.4	6
MQTL2B.2	*Kukri_c23822_580- BS00010318_51*	75.00–76.60	4	9.5	2
MQTL2D.1	*Xbarc90- Xbarc292*	36.69–36.99	3.6	5.9	2
MQTL2D.2	*XksuD23- wsnp_CAP12_c1503_764765*	59.33–72.83	2.7	11.7	2
MQTL3A.1	*Excalibur_c61765_220- Xfba91*	81.10–82.26	2.8	10.3	2
MQTL3B.1	*Xfba190- Xgwm493*	135.66–141.90	8	15.9	3
MQTL3B.2	*BARC131-Xwmc623*	151.83–164.03	4.6	11.4	3
MQTL4A.1	*Ku_c2478_227- Tdurum_contig12696_528*	61.37–64.45	15.3	22	2
MQTL4A.2	*Xwmc96- BS00043286_51*	94.16–97.93	3.6	36.4	2
MQTL4A.3	*Xgwm637- Ra_c74035_586*	105.34–115.46	3.1	11.8	2
MQTL4D.1	*Xcfd84-Xwmc622*	70.41–71.53	8.9	21.3	2
MQTL4D.2	*Xcfd84- Xgwm194*	71.20–84.14	7.5	18.2	3
MQTL5A.1	*RAC875_c53808_1027- Kukri_c95189_124*	480.00–483.36	3.2	14.2	3
MQTL5A.2	*RAC875_c53808_1027- Kukri_c95189_124*	492.41–499.97	3	18.9	2
MQTL5B.1	*wmc149- Kukri_c912_1423*	35.23–37.58	3.3	8.5	2
MQTL5B.2	*Tdurum_contig70554_1004- Tdurum_contig70554_1006*	44.17–44.56	3.1	10	2
MQTL5D.1	*gwm959c- Xwgm583*	32.86–49.95	3.4	21.2	2
MQTL5D.2	*Xgwm583-gbxG083c*	60.92–62.68	9.4	34.9	5
MQTL5D.3	*gbxG083c- Xbarc286*	63.03–64.55	10.8	76.5	5
MQTL5D.4	*Xgwm639-Xcfd12*	67.63–67.69	2.6	21.7	3
MQTL6A.1	*6A-BS00103442_51-Xcdo388*	84.49–88.52	15.3	35.4	4
MQTL6A.2	*Xwmc254- Xwmc59*	180.11–184.49	8.8	16	2
MQTL7A.1	*Ku_c416_1522- Tdurum_contig12832_116*	464.72–472.43	3.4	13.4	5
MQTL7A.2	*Xfbb121-Xcdo545*	476.97–480.91	3.1	15.2	6
MQTL7A.3	*Xwmc158- BS00033782_51*	483.13–484.00	2.7	14.4	6
MQTL7B.1	*Xwmc606- Xwmc323*	136.90–137.95	4.3	21.3	3
MQTL7B.2	*CAP12_c1816_325- Xgwm537*	177.03–179.95	1.5	14.3	3
MQTL7D.1	*Xwmc506- Xwmc450*	19.39–29.83	5.1	7.1	2
MQTL7D.2	*Xmwg710.2- Xgwm130*	41.02–41.93	3.3	21.1	3
MQTL7D.3	*Xgwm1220- BS00027514_51*	55.57–57.52	5	8.7	6
MQTL7D.4	*GENE-4592_95- Xwmc121*	65.11–75.13	4	12.1	3

### Comparison of MQTLs with GWAS-MTAs

The physical coordinates of 39 MQTLs identified in our study were compared to MTAs reported in 10 previous GWAS that comprised a total of 281 MTAs for PM resistance. Among these 39 MQTLs, only 18 were found to be overlapped with GWAS-MTAs (**Supplementary Table S5**). Some MQTLs were found to be co-localized with MTAs available from multiple GWAS; For instance, MQTL6A.1 co-localized with MTAs identified in three different GWAS. Additionally, three MQTLs (viz., 2B.1, 3B.2 and 5A.2) contained at least 3 initial QTL which were co-localized with multiple MTAs reported from two distinct GWAS.

### Co-localization of MQTLs with known PM genes

The identification of the association of known PM genes with individual MQTLs revealed that a total of 2 genes associated with PM resistance, including *Pm2* and *Pm3* were found to be co-localized with 2 MQTLs identified in this study, whereas *Pm8*, *Pm21*, *Pm38* and *Pm41* were found in proximity of MQTLs identified in the present study. (**Supplementary Table S6**).

## Discussion

Multiple QTL mapping studies in wheat related to PM have made significant contributions in advancing our knowledge of the genetic basis of quantitative resistance to PM in wheat (Mohler and Stadlmeier, 2019; Xu et al., 2020; Liu et al., 2021). These studies involve the discovery and mapping of distinct genetic regions associated with resistance against PM, offering valuable insights into the genetic complexity of this trait. However, it is widely acknowledged that QTLs identified using one specific mapping population or parental lines may not always perform effectively in a breeding program involving diverse genetic backgrounds (Yang et al., 2021). This phenomenon highlights the need for a more comprehensive and adaptable approach to harness the potential of these QTLs in wheat improvement programme. Overall, MQTL analysis is a powerful method to gather and synthesize information from multiple QTL mapping studies. It provides a more comprehensive understanding of the genetic basis underlying the trait which further helps to refine QTL positions with reduced CI’s (Welcker et al., 2011). Meta-QTL analysis has been extensively employed to study a plethora of traits, including disease resistance, across various crop species like rice (Kumar and Nagarajah, 2020; Kumar et al., 2020; Shashiprabha et al., 2022), wheat (Marone et al., 2013; Soriano and Royo, 2015; Venske et al., 2019; Zheng et al., 2021; Pal et al., 2022; Saini et al., 2022b), barley (Schweizer and Stein, 2011) and maize (Rossi et al., 2019). In a previous research effort, MQTLs were identified for wheat’s resistance to PM using a limited number of initial QTLs for PM resistance, resulting in the identification of only a small number of MQTLs associated with this trait (Marone et al., 2013). The precision of the meta-analysis findings tends to increase in tandem with the number of initial QTLs employed. It is important to emphasize that a positive relationship typically exists between the precision of results derived from meta-analysis and the number of initial QTLs involved in the analysis (Kumar et al., 2023). Moreover, with the continuous progress in molecular genetics and QTL mapping techniques, there is a consistent discovery and publication of new QTLs. Consequently, it is essential for us to stay updated with these developments to incorporate the latest QTL information into more robust and stable (MQTL) analyses. Hence, in this current study, we conducted a MQTL analysis by integrating QTL data reported in 34 different studies during 1996–2021 on PM resistance and identified 39 MQTLs. to acquire a more profound understanding of how genetic factors regulate resistance to PM in wheat. The first step in the meta-analysis process involved mapping the original QTLs onto a consensus map, which is crucial for identifying common regions of interest through meta-analysis.

Sub-genome B revealed the highest density of genetic markers, thus harboring maximum number of initial QTLs. This finding aligns with previous reports that have explored genetic diversity and the intricate genetic makeup of disease resistance in wheat (Soriano and Royo, 2015; Amo and Soriano, 2022; Pal et al., 2022; Saini et al., 2022b). Conversely, the comparatively low level of genetic variation in the sub-genome D may explain the relatively small number of QTLs detected across various QTL mapping studies which is consistent with previously conducted meta-analyses for disease resistance in wheat that also reported less number of QTLs on sub-genome D (Venske et al., 2019; Liu et al., 2020; Zheng et al., 2021; Pal et al., 2022; Saini et al., 2022a). We believe that our current effort in gathering and analyzing QTL data for PM resistance in wheat represents the most extensive and thorough compilation to date. The highly dense consensus map constructed in our study using four different genetic maps allowed us to identify markers that were closely associated with corresponding MQTLs. The consensus map used in the current study consists of a higher number of markers in comparison to the consensus map utilized in a prior study on MQTL related to PM resistance in wheat, where only 3,618 markers were used and these were obtained by merging only two wheat linkage maps. A higher proportion of QTLs (75.6%) were projected on the consensus map. One potential explanation could be the use of a comprehensive consensus map in the present research. The discovery of 39 MQTLs from the initial pool of 168 QTLs led to a notable decrease by a factor of 2.07 (=10.35/5) in the number of genomic regions or QTLs linked to PM resistance in wheat. Our study stands out as a more up-to-date and comprehensive compilation as compared to a prior meta-analysis on PM resistance (Marone et al., 2013); for several reasons. Firstly, we incorporated a larger dataset, utilizing 222 QTLs from 34 mapping studies, in contrast to 101 QTLs from 20 studies, which has been shown to improve the accuracy of statistical findings. Secondly, we employed a highly dense consensus map with 54,672 markers, as opposed to the earlier study’s use of 3,618 markers. Thirdly, our study integrated a greater number of QTLs (168) into MQTLs due to the use of the dense consensus map. Additionally, we validated 18 MQTLs using GWAS-based MTAs, demonstrating the broader genetic impact of these regions on PM resistance. Moreover, our study employed specific criteria to prioritize hcMQTLs for CG mining, leading to the identification of more promising CGs.

### Candidate genes within the hcMQTLs and their association with PM responses

Candidate gene mining within 9 hcMQTLs revealed 256 unique gene models. Twenty-five promising candidate genes were chosen based on Knet score (**Table 2**). The roles of some genes in conferring resistance to PM is discussed as follows: (i) NBS-LRR domain-containing proteins are also encoded by some cloned *Pm* genes such as *Pm2b*, *Pm60* and *Pm21* that confer PM resistance in wheat (He et al., 2018; Zou et al., 2018; Jin et al., 2022), (ii) Proteins belonging to the protein kinase family are essential components of the defense mechanism in wheat. Receptor-like kinases (RLKs) and plant protection kinases participate in the recognition and initiation of a diverse array of signals connected to various developmental and physiological functions. These include processes related to defense mechanisms as well as beneficial symbiotic interactions (Rentel et al., 2004; Garcia et al., 2012), (iii) Jacalin like lectin domain containing genes have the ability to bind to carbohydrates, recognizing those derived from pathogens or injury during infections (Lannoo and Van Damme, 2014; Esch and Schaffrath, 2017). A mannose-specific JRL (mJRL)-like gene (*TaJRLL1*) which codes for a protein containing two jacalin-like lectin domains has been discovered in wheat. When *TaJRLL1* was introduced into *Arabidopsis thaliana*, resistance towards two fungal pathogens, *F*. *graminearum* and *Botrytiscinerea* was enhanced. The levels of jasmonic acid (JA) and salicylic acid (SA) showed a substantial increase in transgenic Arabidopsis plants. These results suggest that *TaJRLL1* could be a component of the SA and JA dependent defense signaling pathways (Xiang et al., 2011), (iv) Glycoside hydrolases (GHs) are the most prevalent and widely distributed class of enzymes in fungi, as observed in various studies and their primary function is to enzymatically break down the glycosidic bonds between carbohydrate molecules or between carbohydrates and non-carbohydrate groups (Zhou et al., 2013). One study discovered that GH proteins are recognized by the leucine-rich-repeat receptor-like protein in the dicot plant *Nicotiana benthamiana*. Heterologous expression of this receptor in wheat, made it responsive towards GH proteins, resulting in an increased resistance to *F*. *graminearum* (a fungal pathogen) and lower levels of the mycotoxin deoxynivalenol in wheat grains (Wang et al., 2023a), (v) Transcription factors like GRAS, MYB and MADS-box play an important role in plant disease resistance. 1R-MYB transcription factor was reported to play key role in disease resistance against stripe rust fungus in wheat (Zhang et al., 2015). Studies by Zhang et al. (2016) and Castelán-Muñoz et al. (2019) have reported that members of the MADS-box gene family participate in the control of both biotic and abiotic stress reactions, which indicates a potential role in stress response. Several MADS-box genes were activated and displayed varying levels of expression after inoculation of wheat spikes with FHB indicating their potential defensive roles against Fusarium infections (Kugler et al., 2013), (vi) The gene *TaCYP72A* which encodes cytochrome P450, plays a significant role enhancing host resistance to Fusarium head blight (FHB) in wheat (Gunupuru et al., 2018), (viii) Synaptobrevin domain containing proteins-TaSYP137 and TaVAMP723, the SNAREs proteins containing streptobrevin and longin domains have been reported to reduce resistance to *Blumeria graminis f.* sp. *Tritici* in wheat (Wang et al., 2023b).

**Table 2:** Most promising candidate genes associated with powdery mildew resistance.

hcMQTL	Gene Ids	Gene Position (bp)	Knet Score	Functional description
MQTL1A.1	*TraesCS1A03G0022100*	4497200- 4503419	184.25	NB-ARC
MQTL1A.1	*TraesCS1A03G0025200*	5645211- 5650510	39.16	NB-ARC
MQTL1A.1	*TraesCS1A03G0026100*	6007940- 6012646	16.07	NB-ARC
MQTL1A.2	*TraesCS1A03G0027400*	6293918- 6294835	21.76	Leucine-rich repeat domain superfamily
MQTL1A.2	*TraesCS1A03G0032400*	7311700- 7312269	139.08	Transcription factor, MADS-box
MQTL2B.1	*TraesCS2B03G0094000*	22781478- 22785668	32.12	NB-ARC
MQTL2B.1	*TraesCS2B03G0098400*	23575924- 23578715	24.98	Glycoside hydrolase, family 19, catalytic
MQTL2B.1	*TraesCS2B03G0098900*	23596456- 23597365	25.95	Jacalin-like lectin domain
MQTL3B.1	*TraesCS3B03G0631000*	381767017- 381759928	23.74	SANT/Myb domain
MQTL3B.2	*TraesCS3B03G1090700*	681100681- 681097254	151.3	Transcription factor GRAS
MQTL3B.2	*TraesCS3B03G1087500*	679645157- 679642968	21.6	Transcription factor, MADS-box
MQTL4D.2	*TraesCS4D03G0817500*	503968880- 503969956	16.86	BTB/POZ domain
MQTL4D.2	*TraesCS4D03G0815200*	503615478- 503621888	13.89	EF-hand domain
MQTL4D.2	*TraesCS4D03G0814000*	503355740- 503359344	13.89	Palmitoyl protein thioesterase
MQTL5A.1	*TraesCS5A03G0006500*	2408514- 2406435	32.12	Synaptobrevin
MQTL5A.1	*TraesCS5A03G0009300*	3177753- 3175334	47.72	Cytochrome P450
MQTL5A.1	*TraesCS5A03G0009400*	3197691- 3194780	15.6	Cytochrome P450
MQTL5A.1	*TraesCS5A03G0009500*	3218868- 3215100	19.86	3-beta hydroxysteroid dehydrogenase/isomerase
MQTL5A.1	*TraesCS5A03G0009700*	3294536- 3281861	19.86	Terpene synthase, conserved site
MQTL5A.1	*TraesCS5A03G0010300*	3374577- 3370704	33.82	Protein kinase domain
MQTL6A.1	*TraesCS6A03G0385400*	149962969- 149954398	9.1	T-complex 11
MQTL6A.1	*TraesCS6A03G0386400*	150987049- 150982707	10.56	Fumarylacetoacetase
MQTL7D.4	*TraesCS7D03G0436800*	151917055- 151915776	13.89	Plant peroxidase
MQTL7D.4	*TraesCS7D03G0437800*	152121744- 152120457	10.08	Glycoside hydrolase family 16
MQTL7D.4	*TraesCS7D03G0438700*	152949088- 152947488	15.25	NAC domain

### Concordance between MQTLs and known major genes

While there is a record of over 100 PM resistance genes/alleles within 63 loci (*Pm1*-*Pm66*), only a handful of them have been cloned and thoroughly characterized. These include *Pm1a, Pm2a, Pm2b, Pm3b, Pm5e, Pm8, Pm17, Pm21, Pm24, Pm38, Pm41, Pm46*, and *Pm60*, while the majority remain uncharacterized (Yahiaoui et al., 2004; Krattinger et al., 2009; Moore et al., 2015; Sánchez-Martín et al., 2016; He et al., 2018; Singh et al., 2018; Xing et al., 2018; Zou et al., 2018; Zhang et al., 2019; Li et al., 2020; Lu et al., 2020; Xie et al., 2020; Hewitt et al., 2021; Jin et al., 2022). Except for *Pm24*, *Pm38*, and *Pm46*, which code for a tandem kinase gene known as WTK3, an ABC transporter, and a hexose transporter, respectively, most of the characterized PM resistance genes in wheat are categorized as NLR proteins and exhibit specificity to distinct pathogen races (Mapuranga et al., 2022). As many as 2 PM resistance genes, including *Pm2* and *Pm3* were found to share the same genomic locations as overlap with 2 MQTLs that were identified in this study. For instance, MQTL1A.1 co-localized with *Pm3a* and *Pm3b* and MQTL5D.1 co-localized with both *Pm2a* and *Pm2b* genes conferring resistance to PM in wheat, thus confirming the effectiveness of employing a highly saturated consensus map in MQTL analysis. Furthermore, 4 PM (*Pm8*, *Pm21*, *Pm38* and *Pm41*) genes were found in proximity to the MQTLs identified in the present study.

Recently, Jin et al. (2022) established that the PM resistance genes *Pm2b* was located within the same genomic region as *Pm2a* and *PmCH1357*. However, these genes exhibited distinct resistance profiles, suggesting that *Pm2* exhibits a diverse resistance spectrum among its multiple alleles. The researchers also identified a transcription factor called *TaWRKY76-D* as an interacting partner of *Pm2b*, and this interaction relied on the NB domain of *Pm2b* and the WRKY domain of *TaWRKY76-D*. Interestingly, *TaWRKY76-D* was found to have a negative regulatory effect on PM resistance in wheat. Additionally, the team developed a specific KASP marker known as K529, which offers the advantages of high-throughput and high-efficiency for the detection of *Pm2b*. In the current study, MQTL1A.1 was discovered to co-localize with both *Pm3a* and *Pm3b*, suggesting a connection between these genes and reinforcing the fact that 10 alleles providing race-specific resistance to PM exist at the *Pm3* locus in hexaploid wheat.

### Assessing the effectiveness of MQTL through GWAS

GWAS is a promising approach for exploring intricate traits, leveraging both recent and past recombination events within the association panel, thereby enabling precise mapping of these traits (Bush and Moore, 2012; Saini et al., 2022c; Singh et al., 2023; Reddy et al., 2023). The emergence of cost-effective, high-throughput sequencing technologies have simplified the discovery of MTAs associated with various disease resistance traits using genome-wide variants (Kumar et al., 2020; Alemu et al., 2021; Tomar et al., 2021; Nannuru et al., 2022; Vikas et al., 2022; Pradhan et al., 2023). In this study, approximately 46% (18 out of 39) of the identified MQTLs were confirmed through associations with MTAs related to PM resistance. The limited verification of MQTLs by GWAS-MTAs may be attributed to various factors or reasons. Firstly, neither of these methods (MQTL or GWAS) comprehensively captures all the genetic diversity inherent within the crop species. Secondly, there is a notable disparity in the genetic materials utilized between these two approaches. Furthermore, it is important to note that GWAS primarily targets the identification of common or frequent genetic variants, typically those with a minor allele frequency exceeding 5%. Additionally, environmental factors can significantly influence trait expression, and if not adequately controlled for, they may obscure associations between markers and traits. On the contrary, linkage-based interval mapping studies excel in detecting rare alleles that significantly influence the phenotype.

The stability and reliability of MQTLs could be enhanced when they are corroborated by MTAs identified through multiple GWAS studies and encompass numerous QTLs derived from different interval mapping studies. In our study, four such MQTLs (MQTL2B.1, MQTL3B.2, MQTL6A.1, MQTL5A.2) with at least 2 initials QTLs from different studies with reduced CI (95%) (average CI< 6cM) were verified with multiple MTAs obtained from different GWAS. These meta-QTLs have immense potential and can be regarded as valuable candidates for use in marker-assisted breeding (MAB) initiatives aimed at enhancing PM resistance in wheat.

## Conclusions

Our current could identify the most stable and reliable QTLs associated with PM resistance in wheat, providing insight into the intricate quantitative genetic structure of PM resistance. Out of the 39 MQTLs which were identified, 9 hcMQTLs were selected for further investigation to identify the underlying CGs. A total of 256 unique candidate genes were identified within the hcMQTLs, with 25 promising candidates belonging to various gene families known to play roles in disease resistance, such as NBS-LRR, protein kinases, jacalin-like lectins, glycoside hydrolases, and transcription factors. The co-localization of MQTLs with known powdery mildew resistance genes (*Pm2, Pm3, Pm8, Pm21, Pm38*, and *Pm41*) validated the effectiveness of the MQTL analysis. The obtained MQTLs would be useful for further understanding of the molecular mechanisms of PM resistance and for the development of PM-resistant wheat varieties. Additionally, information pertaining to markers flanking the MQTLs can be integrated into genomic selection models, thereby, improving the accuracy of PM resistance through more accurate estimates of genomic estimated breeding values (GEBVs). In the future, breeders have the opportunity to enhance PM resistance trait in wheat by utilizing the most promising MQTLs, specifically 1A.1, 1A.2, 2B.1, 3B.1, 3B.2, 4D.2, 5A.1, 6A.1 and 7D.4 as identified in this research. The candidate gene underlying these MQTLs could bevalidated for marker-assisted breeding programs aimed at enhancing PM resistance in wheat.

## Data availability statement

The original contributions presented in the study are included in the article/**Supplementary Material**. Further inquiries can be directed to the corresponding authors.

## Author contributions

DS: Writing – review & editing, Writing – original draft, Software, Data curation, Conceptualization. NB: Writing – review & editing, Software, Conceptualization. AK: Writing – original draft, Software, Data curation. DKS: Writing – review & editing, Supervision, Conceptualization. AS: Writing – original draft, Data curation. AY: Writing – original draft, Data curation. RM: Writing – review & editing, Supervision. AKS: Writing – review & editing, Supervision. VKV: Writing – review & editing, Supervision. GS: Writing – review & editing, Supervision. DK: Writing – review & editing, Supervision, Conceptualization.
